# *Helicobacter pylori* infection is not associated with portal hypertension-related gastrointestinal complications: A meta-analysis

**DOI:** 10.1371/journal.pone.0261448

**Published:** 2022-01-21

**Authors:** Yu Kyung Jun, Ji Won Kim, Byeong Gwan Kim, Kook Lae Lee, Yong Jin Jung, Won Kim, Hyun Sun Park, Dong Hyeon Lee, Seong-Joon Koh

**Affiliations:** 1 Department of Internal Medicine and Liver Research Institute, Seoul National University College of Medicine, Seoul, Korea; 2 Laboratory of Intestinal Mucosa and Skin Immunology, SMG-SNU Boramae medical Center, Seoul, Korea; 3 Department of Internal medicine, Division of Gastroenterology, SMG-SNU Boramae medical Center, Seoul, Korea; 4 Department of Dermatology, SMG-SNU Boramae medical Center, Seoul, Korea; PLOS, UNITED KINGDOM

## Abstract

Despite the importance of *Helicobacter pylori* infection and portal hypertension (PH)-associated gastrointestinal (GI) diseases, such as esophageal varices and portal hypertensive gastropathy (PHG), the impact of *H*. *pylori* infection on PH-related GI complications has not yet been elucidated. This meta-analysis investigated the association between *H*. *pylori* infection and the risk of PH-related GI complications. An electronic search for original articles published before May 2020 was performed using PubMed, EMBASE, and the Cochrane Library. Independent reviewers conducted the article screening and data extraction. We used the generic inverse variance method for the meta-analysis, and Begg’s rank correlation test and Egger’s regression test to assess publication bias. A total of 1,148 cases of *H*. *pylori* infection and 1,231 uninfected controls were included from 13 studies. *H*. *pylori* infection had no significant association with esophageal varices [relative risk (RR) = 0.96, 95% confidence interval (CI) = 0.87–1.06 for all selected studies; RR = 0.95, 95% CI = 0.84–1.07 for cohort studies; odds ratio (OR) = 0.96, 95% CI = 0.60–1.54 for case-control studies]. Although *H*. *pylori* infection was significantly associated with PHG in case-control studies [OR = 1.86, 95% CI = 1.17–2.96], no significant differences were found in the cohort studies [RR = 0.98, 95% CI = 0.91–1.05] or all studies combined [RR = 1.18, 95% CI = 0.93–1.52]. In conclusion, *H*. *pylori* infection was not associated with the risk of PH-related GI complications. Clinicians should carefully treat cirrhotic patients with PH-related GI complications, regardless of *H*. *pylori* infection.

## Introduction

Liver cirrhosis is a disease characterized by chronic fibrosis and dysfunction of the liver. In cirrhotic patients, structural abnormalities, including hepatic vascular resistance, and functional defects, including endothelial dysfunction and increased hepatic vascular tone, increase hepatic resistance to portal blood flow and elevate portal pressure. Alterations in intrahepatic hemodynamics provoke splanchnic vasodilation as an adaptive response. As cirrhosis progresses, splanchnic vasodilation becomes too intense to have serious effects on systemic circulation. Varices and portal hypertensive gastropathy (PHG) can develop as complications of portal hypertension (PH) in patients with cirrhosis. Varices and PHG are observed in approximately 50% and 3–14% of cirrhotic patients, respectively, and can be the main causes of massive bleeding, leading to hemodynamic instability and a life-threatening disease course [1]. Therefore, controlling PH-associated GI diseases in patients with liver cirrhosis is a great challenge.

*Helicobacter pylori* can colonize the acidic gastric mucosal surface. Gastric colonization by *H*. *pylori* can induce peptic ulcer disease, atrophic gastritis, gastric cancer, and mucosa-associated lymphoid tissue lymphoma. *H*. *pylori* also contributes to non-GI disorders, such as iron-deficient anemia, vitamin B12 deficiency, and idiopathic thrombocytopenic purpura. Extragastric diseases that were previously considered to be independent of *H*. *pylori* have recently been found to be related to *H*. *pylori* infection [2,3]. Multiple liver diseases, such as chronic viral hepatitis and nonalcoholic fatty liver disease (NAFLD), are also known to be associated with *H*. *pylori* infection [4–6]. Many studies have demonstrated a positive relationship between *H*. *pylori* infection and cirrhosis, regardless of PH-related GI complications [7]. Moreover, a cirrhotic patient with a higher Model for End-stage Liver Disease (MELD) score can show higher positivity for *H*. *pylori* infection [8]. *H*. *pylori* eradication therapy can also improve hyperammonemia in patients with cirrhosis [9]. Although there are several articles regarding the presence of *H*. *pylori* infection in cirrhotic patients with varices or PHG, these show controversial results concerning the relationship between *H*. *pylori* infection and PH-related GI complications [10–20]. Therefore, we aimed to integrate articles dealing with the relationship between *H*. *pylori* infection and PH-related complications in patients with cirrhosis.

## Materials and methods

### Data sources and searches

Two authors (Y.K.J. and D.H.L.) independently carried out a comprehensive systematic search for published articles from inception to May 2020 using PubMed, EMBASE, and the Cochrane Library. The search was limited to human studies without language restrictions. Search terms included “*Helicobacter*,” “*pylori*,” “*pyloridis*,” “*Helicobacter pylori*,” or “*Campylobacter pylori*” and “liver cirrhosis,” “liver fibrosis,” “hepatic fibrosis,” “liver failure,” “hepatic failure,” “esophageal varix,” “varix,” “variceal,” or “portal hypertensive gastropathy” which are described in **S1 Text**. We manually searched major international gastroenterology and hepatology conference abstracts and the references of the selected articles. Additionally, we found articles through internet searches to identify further relevant studies. The current study was conducted following the Preferred Reporting Items for Systematic review and Meta-Analysis protocols (PRISMA) guidelines [21].

### Selection criteria

Studies that met the following eligibility criteria were included: 1) observational studies, including cohort and case-control studies; 2) studies that evaluated the relationship between *H*. *pylori* infection and PH-related GI complications; 3) appropriate diagnostic tests for *H*. *pylori* infection conducted prior to the evaluation of PH-related GI complications; and 4) available raw data reported in the comparison arms.

Studies that did not include original articles were excluded. Also, studies were excluded if they were case studies, had no control group, or were not performed on human subjects. We excluded articles that did not explicitly state institutes or hospitals where the research was performed. When duplicated publications were identified, we included the most recent and thorough articles.

### Data extraction and outcomes

Data extraction was conducted independently by two authors (Y.K.J. and D.H.L.). The following data were collected from the included studies: first author, year of publication, study design, demographic and clinical information of the study patients (age, sex, ethnicity, and etiology of liver cirrhosis), methods of *H*. *pylori* detection, and outcome results in patients with or without *H*. *pylori* infection.

The retrieved articles were independently reviewed by fully qualified investigators (Y.K.J., D.H.L., and S.J.K.), and disagreements were resolved through discussions among investigators. The Newcastle-Ottawa quality assessment Scale (NOS), which was developed as a judgment tool for the quality of nonrandomized studies such as case-control or cohort studies in meta-analyses, was used to evaluate the quality of the selected studies [22]. The questions in the NOS questionnaire were categorized as items of selection, comparability, and exposure, and were scored using stars. Studies scoring 8–9 stars were categorized as high-quality, 6–7 stars as moderate-quality, and 0–5 stars as low-quality [23].

### Statistical analysis

All statistical analyses were performed using Stata software (version 15.0; Stata Corporation, College Station, TX) and R (version 4.0.1; The R Project for Statistical Computing, Vienna, Austria). The odds ratio (OR), relative risk (RR), and 95% confidence interval (CI) were considered to be the effect sizes. We used the unadjusted OR and RR values calculated from the raw data. Considering the low incidence of PH-related GI complications in *H*. *pylori*-infected patients, we assumed that the ORs were similar to RRs. The generic inverse variance method was used to combine the results across studies. Heterogeneity among studies was assessed using the *I*^2^ statistics test considering low heterogeneity (<25%), moderate heterogeneity (25–75%), and high heterogeneity (>75%). Subgroup analyses based on NOS information were also performed. The probability of publication bias was assessed using the Begg’s rank correlation test and Egger’s regression test. Statistical significance was set at *P* <0.05.

## Results

### Literature search

**Fig 1:**
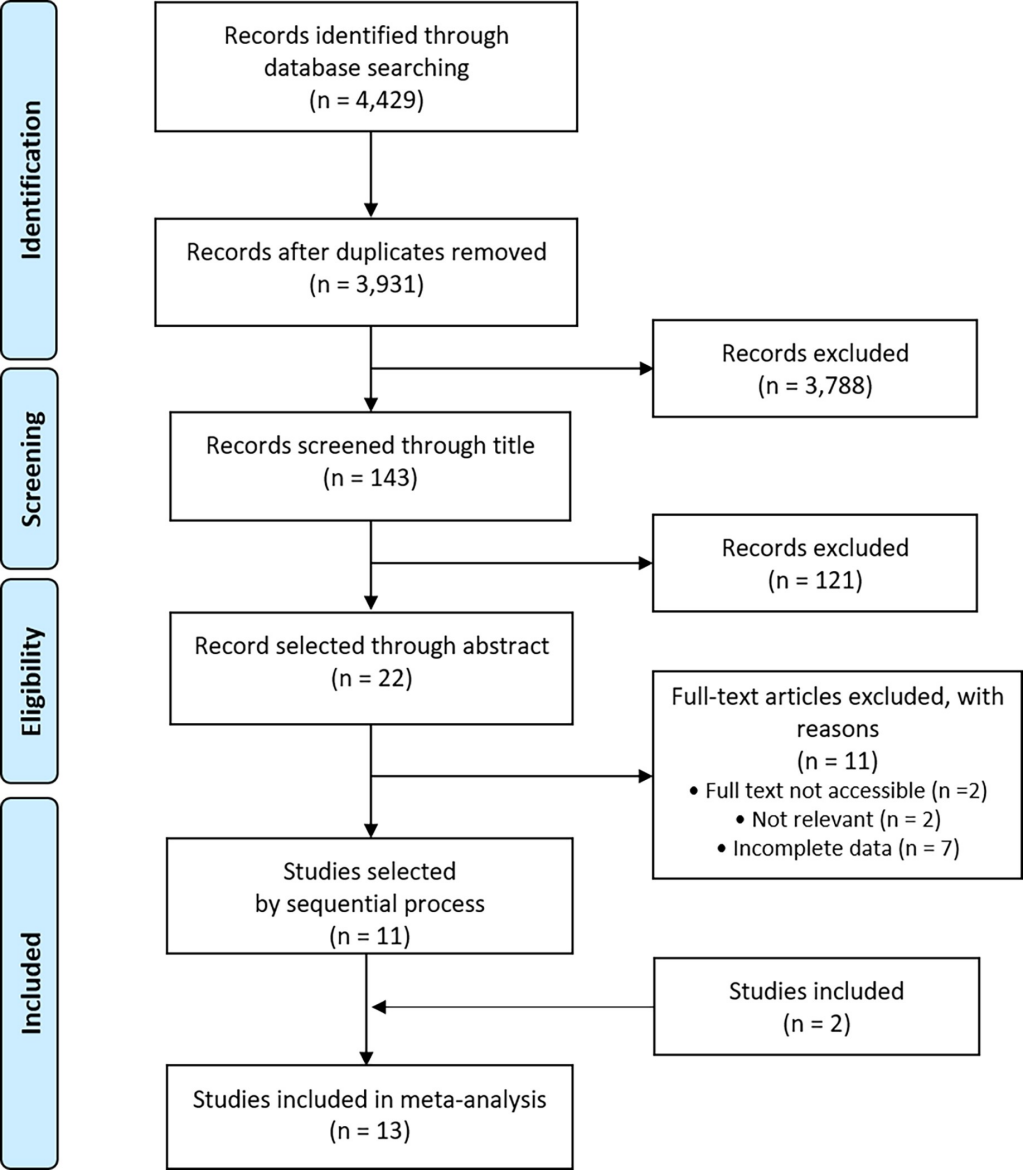
Flow diagram of study selection. n, number.

Through the database search, 4,429 potentially eligible studies were identified (752 journals from PubMed, 3,381 journals from EMBASE, and 296 journals from the Cochrane library). We excluded 498 duplicated studies. After evaluating study titles and abstracts, 3,788 studies and 121 studies were excluded. After a full-length article review, 11 studies were excluded, and other 11 studies were included. Among these excluded studies, 2 studies were not accessible, 2 studies did not have relevant content, and 7 studies did not report the complete outcomes of interest. Two studies were identified after additional searches. Finally, 13 studies (8 cohort studies and 5 case-control studies) were included in the meta-analysis [10–13,15–19,24,25]. The literature search and selection flow diagram are described in **Fig 1**.

### Study characteristics and quality

The baseline characteristics and quality assessments of the 13 trials are summarized in **Table 1**. A total of 2,379 patients were included in the meta-analysis. *H*. *pylori*-specific serum immunoglobulin G was most frequently used for the diagnosis of *H*. *pylori* infection [10,12,14,17,19,25]. The common causes of cirrhosis were viral hepatitis and alcoholic liver disease. Two articles [11,26] did not include patients with cirrhosis caused by hepatitis virus infection. Five articles gave the mean age of *H*. *pylori*-positive patients and *H*. *pylori*-negative patients, respectively [12,13,16,19,20]. Mean age of *H*. *pylori*-positive patients was 54.1 ± 10.8 years and that of *H*. *pylori*-negative patients was 54.8 ± 9.4 years. There was no significant age difference according to *H*. *pylori* infection. The median NOS score of the trials that were included was 7 (range 5–8), and we considered 5 of 13 trials to be of high methodological quality (**S1 and S2 Tables**).

**Table 1:** Main characteristics of all studies in the meta-analysis (ordered by publication year and study design).

Study	Country	Study design	Diagnostic tool for *H*. *pylori*	Etiology of LC^a^	End points	Male (%)	Mean age	*H*. *pylori*-infected n (+ vs. -)	NOS
**Wu *et al*., 1995 [10]**	Taiwan	Case-control	Serum IgG	HBV, HCV, HBV-HDV	EV	73.3	51.5	82 vs. 120	6
**Balan *et al*., 1996 [11]**	UK	Cohort	Histology	Alc, PBC	PHG	58	53^a^	20 vs. 30	8
**Bahnacy *et al*., 1997 [25]**	Hungary	Case-control	Serum IgG	Alc, Hep, PBC, PVB	PHG	73.3	49.1	23 vs. 67	5
**Tsai, 1998 [12]**	Taiwan	Cohort	Serum IgG, histology, CLO	HBV, HCV, Alc, HBV-HCV, HBV-HDV	EV	66.2	54.4	99 vs. 22	8
**McCormick *et al*., 1999 [24]**	UK	Cohort	Histology	Alc, PVB, Schi, congenital	PHG	53.8	51.5	22 vs. 71	7
**Yeh *et al*., 2001 [13]**	Taiwan	Cohort	13C-UBT	HBV, Alc, HCV, HBV-HCV	EV, PHG	72.5	57.4	57 vs. 52	8
**Chen *et al*., 2002 [14]**	Taiwan	Case-control	Serum IgG	HBV, HCV, Alc	EV	76.6	64.8^b^	42 vs. 57	5
**Arafa *et al*., 2003 [19]**	Japan	Cohort	13C-UBT, Serum IgG	HBV, HCV, Alc	PHG	78.3	63	31 vs. 29	8
**Urso *et al*., 2006 [15]**	Italy	Cohort	Histology, 13C-UBT	HCV	PHG	58.7	69.7	26 vs. 83	7
**Abbas *et al*., 2014 [16]**	Pakistan	Cohort	Histology, PCR	HCV, HBV, HBV-HDV	EV, PHG	65.7	50.3	87 vs. 53	7
**Sathar *et al*., 2014 [17]**	India	Case-control	Serum IgG	Alc, HBV, HCV, AIH	PHG	68.55	53.45	50 vs. 90	6
**Huang and Cui, 2017 [18]**	China	Case-control	13C-UBT	HBV	PHG	59.9	52.5	339 vs. 269	5
**Abdel-Razik *et al*., 2020 [20]**	Egypt	Cohort	fecal antigen	HBV, HCV	EV, PHG	72.4	54.1	270 vs. 288	8

*H*. *pylori*, *Helicobacter pylori*; LC, liver cirrhosis; n, number of patients; NOS, Newcastle-Ottawa scale; UK, United Kingdom; IgG, immunoglobulin G; CLO, *Campylobacter*-like organism; UBT, urea breath test; Alc, alcohol; PBC, primary biliary cholangitis; Hep, viral hepatitis; PVB, portal vein block; Schi, schistosomiasis; AIH, autoimmune hepatitis; EV, esophageal varices; PHG, portal hypertensive gastropathy.

^a^Etiologies are sorted by largest number of patients.

^b^Median age.

### The relationship between *H*. *pylori* infection and the risk of PH-related GI complications

**Fig 2:**
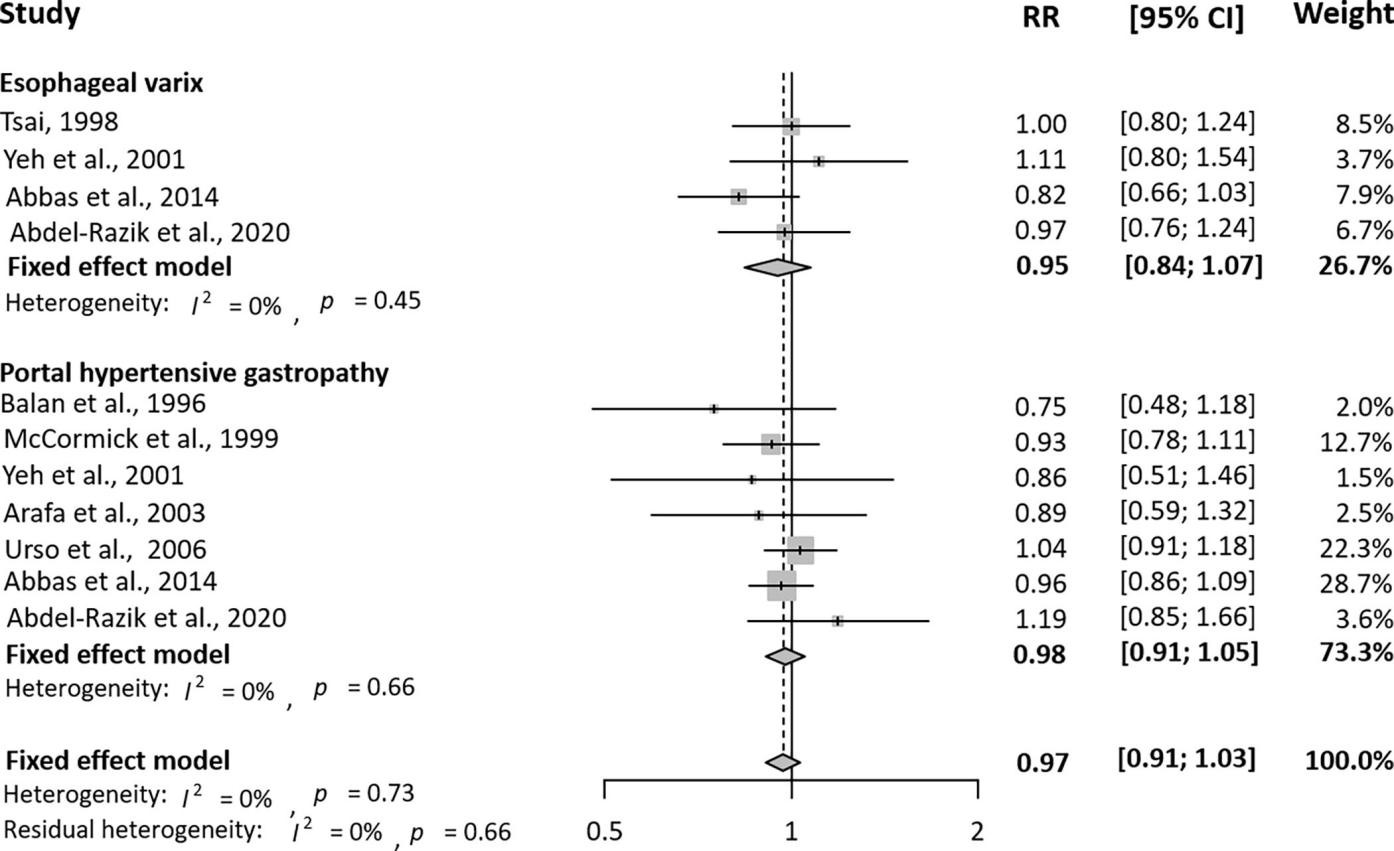
Unadjusted relative risk and 95% confidence interval of portal hypertension-related gastrointestinal complications by *Helicobacter pylori* infection in eight cohort studies. RR, relative risk; CI, confidence interval.

**Fig 3:**
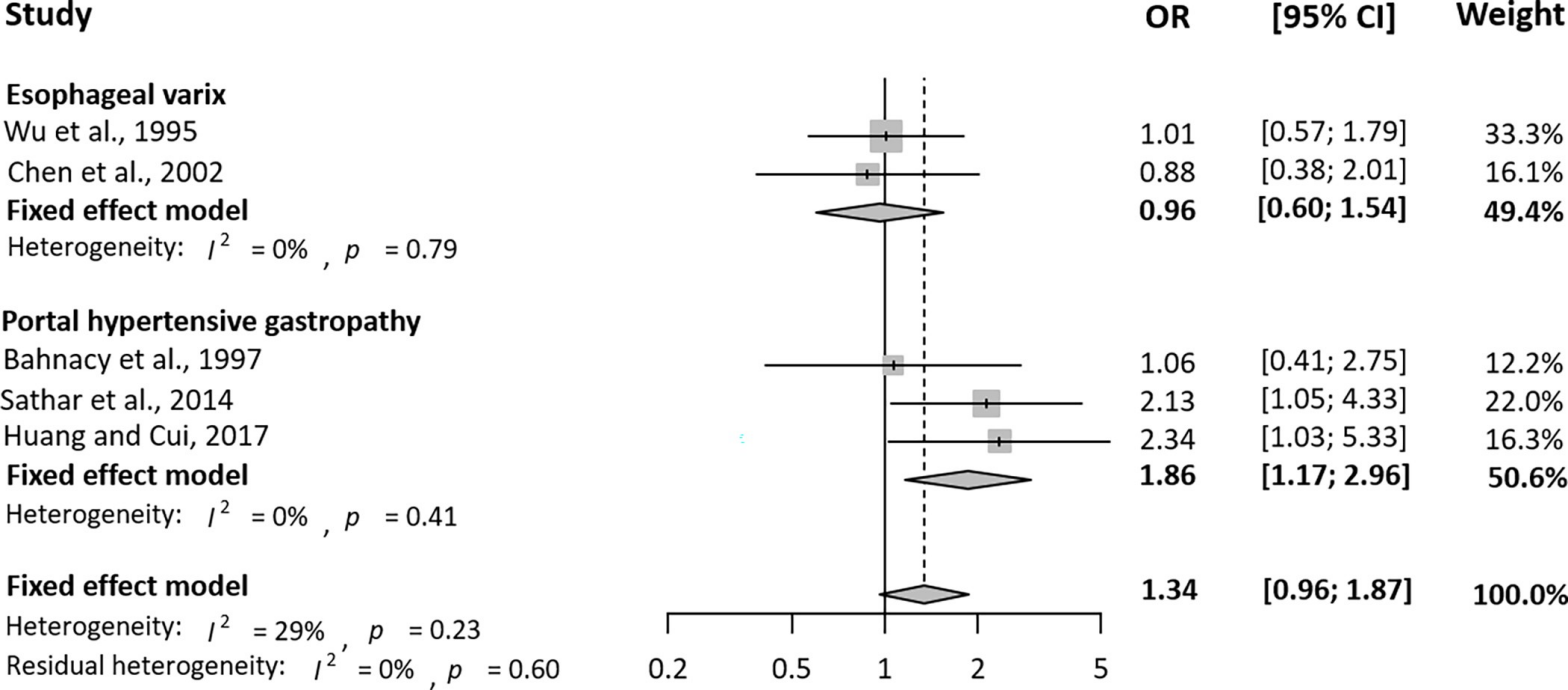
Unadjusted odds ratio and 95% confidence interval of portal hypertension-related gastrointestinal complications by *Helicobacter pylori* infection in five case-control studies. OR, odds ratio; CI, confidence interval.

The meta-analysis results of the association between *H*. *pylori* infection and PH-related GI complications are shown in **Table 2**. *H*. *pylori* infection was not significantly associated with esophageal varices (RR = 0.96, 95% CI = 0.87–1.06). Similarly, there was no association between *H*. *pylori* infection and PHG (RR = 1.01, 95% CI = 0.94–1.08). Subgroup analyses based on information on the study design and NOS were conducted. **Figs 2** and **3** show the unadjusted RR/OR and 95% CI of PH-related GI complications by *H*. *pylori* infection in cohort studies and case-control studies, respectively. There was no meaningful relationship between esophageal varices and *H*. *pylori* in either cohort (RR = 0.95, 95% CI = 0.84–1.07) or case-control studies (OR = 0.96, 95% CI = 0.60–1.54). *H*. *pylori* infection was shown to be unrelated to PHG in cohort studies (RR = 0.98, 95% CI = 0.91–1.05), but in case-control studies of the relationship between *H*. *pylori* and PHG, it was noted that there was some relationship between *H*. *pylori* infection and PHG (OR = 1.86, 95% CI = 1.17–2.96).

**Table 2:** Meta-analysis of relationship between *Helicobacter pylori* infection and portal hypertension-related gastrointestinal complications.

PH-related GI complications	Study design	Studies, N	Heterogeneity		M	Effect size			
			I^2^ (%)	P_H_		RR	OR	95% CI	P_ES_
**Esophageal varix**	Cohort study	4	0	0.4460	F	0.95	–	0.84–1.07	0.4188
	Case-control study	2	0	0.7919	F	–	0.96	0.60–1.54	0.8738
	Total	6	0	0.7258	F	0.96	–	0.87–1.06	0.446
			0	0.7236		–	0.93	0.73–1.19	0.5772
**Portal hypertensive gastropathy**	Cohort study	7	0	0.6557	F	0.98	–	0.91–1.05	0.5489
	Case-control study	3	0	0.4112	F	–	1.86	1.17–2.96	0.0092
	Total	10	29.5	0.1737	F	1.01	–	0.94–1.08	0.8825
			24.6	0.2173		–	1.18	0.93–1.52	0.2177

PH, portal hypertension; GI, gastrointestinal; N, number; P_H_, *p*-value for heterogeneity; M, model for meta-analysis; F, fixed-effect model; RR, relative risk; OR, odds ratio; P_ES_, *p*-value for effect size.

**Fig 4:**
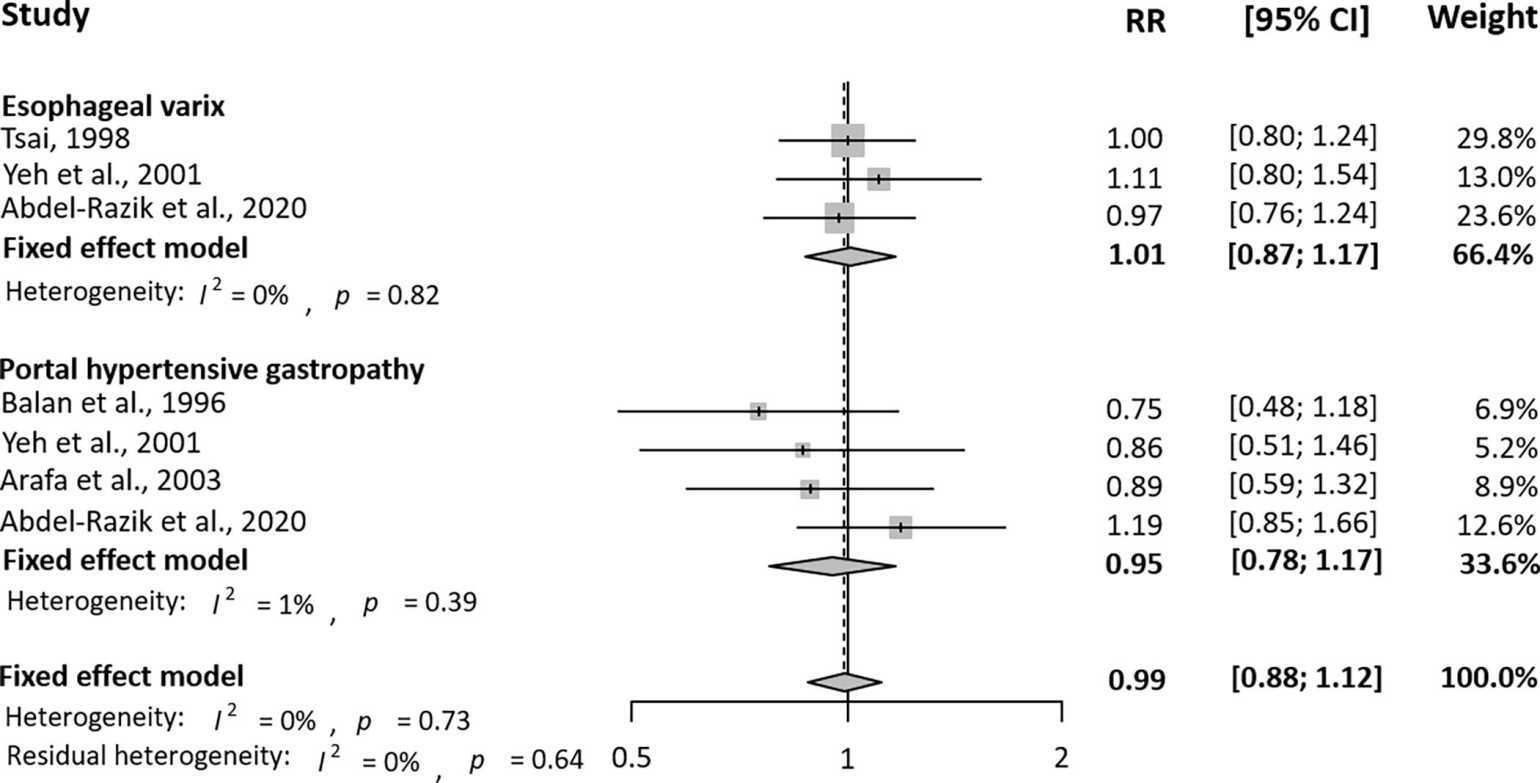
The relationship between portal hypertension-related gastrointestinal complications and *Helicobacter pylori* infection in highly qualified studies. RR, relative risk; CI, confidence interval.

For subgroup analysis, we selected highly qualified articles with NOS>7 and analyzed them in **Fig 4**. Five cohort studies were included in the subgroup analysis, and all case-control studies were excluded. There was no significant correlation between *H*. *pylori* infection and PH-related GI complications in patients with cirrhosis (RR = 0.99, 95% CI = 0.88–1.12).

### Heterogeneity analysis, sensitivity analysis, and publication bias

There was no heterogeneity found (*P_H_*>0.10) in the pooled estimates of cirrhotic patients with varices. The corresponding pooled ORs were not significantly influenced by omitting any single study, as shown in **S3 Table**. Publication bias in this study was unremarkable. All *P*-values obtained from *I*^2^ (*P_H_*), Begg’s (*P_Begg_*) and Egger’s methods (*P_Egg_*) are shown in **Tables 2** and **3**.

**Table 3:** Publication bias of studies conducted by Begg’s rank correlation test and Egger’s regression test.

PH-related GI complication	Study design	Studies, N	Publication bias	
			P_Begg_	P_Egg_
**Esophageal varix**	Cohort study	4	0.497	0.405
	Case-control study	2	0.317	–
	Total	6	0.573 (RR) 0.573 (OR)	0.762 (RR) 0.425 (OR)
**Portal hypertensive gastropathy**	Cohort study	7	0.453	0.459
	Case-control study	3	0.602	0.423
	Total	10	0.655 (RR) 0.128 (OR)	0.480 (RR) 0.220 (OR)

* PH, portal hypertension; GI, gastrointestinal; N, number; P_Begg_, *p*-value for Begg’s test; P_Egg_, *p*-value for Egger’s test.

## Discussion

The relationship between *H*. *pylori* infection and PH-related GI complications is not yet well-understood, although a meta-analysis performed by Feng *et al*. [27] revealed a high prevalence of *H*. *pylori* infection in patients with cirrhosis [10,11]. Unlike previous meta-analyses, we analyzed whether *H*. *pylori* infection contributes to PH-related GI complications in patients with liver cirrhosis. In the present study, it was found that PH-associated GI complications were not related to gastric colonization by *H*. *pylori*. We believe that this study is the first study to demonstrate the association between *H*. *pylori* infection and PH-related GI complications.

Many studies have suggested a strong relationship between *H*. *pylori* infection and liver cirrhosis [18,28–31]. The rate of *H*. *pylori* infection was higher in chronic hepatitis patients than in healthy controls, and cirrhotic patients with *H*. *pylori* infection had poorer outcomes than those without. The positivity of the *H*. *pylori* cytotoxin-associated gene A (CagA) gene in liver tissue is associated with the severity of hepatic fibrosis. Moreover, *H*. *pylori* infection can lead to and aggravate NAFLD [6,32]. The incidence of hepatocellular carcinoma increases when chronic hepatitis C patients are co-infected with *H*. *pylori* [30]. *H*. *pylori* eradication greatly improves cirrhosis and cirrhotic complications [20,33,34].

The relationship between PH-related GI complications and *H*. *pylori* infection in cirrhotic patients is different, although many published studies have demonstrated a positive relationship between liver cirrhosis and *H*. *pylori* infection. Vasodilation mainly contributes to the development of PH by increasing resistance to portal flow and expansion of collateral circulation [35]. Nitric oxide (NO), a vasodilator in PH, provokes an anti-inflammatory response against bacterial infection and converts the viable *H*. *pylori* spiral form into an inviable coccid form [36,37]. *H*. *pylori* can survive in acidic environment, and its growth was limited in neutral pH [38,39]. The range of gastric pH in the general population is 0.3–2.9 [40]. However, gastric pH in PHG patients is higher than that in the general population, and the more severe the PHG, the lesser the gastric acidity [41]. Gastric vascular congestion in PHG may suppress *H*. *pylori* colonization. The severity of gastric vascular congestion is not associated with the possibility of H. pylori infection [42–44].

*H*. *pylori* infection can be suppressed in patients with PH, including varix and PHG, although *H*. *pylori* infection can increase in patients with cirrhosis and result in poor prognosis. Our recent study revealed an insignificant relationship between PH-related GI complications and *H*. *pylori* infection as in the previous studies. Analysis using three case-control studies showed the possibility of a positive relationship between PH-related GI complications and *H*. *pylori* infection. However, case-control studies are less reliable than cohort studies in terms of evidence-based medicine. Moreover, the selected case-control studies had a lower quality than cohort studies. Subgroup analysis, including only articles with NOS>7, failed to show a meaningful relationship between *H*. *pylori* infection and PH-related GI complications. However, in a cohort study of patients with cirrhosis, patients with *H*. *pylori* infection had a poor prognosis, including PH-related GI complications, as compared with those without [20].

Although there have been previous studies on the relationship between *H*. *pylori* infection and PH-related complications, this meta-analysis may be the first to establish a comprehensive and reliable analysis. Prior studies covered only one or a few etiologies of chronic liver disease, but this study handles multiple etiologies of cirrhosis, including viral hepatitis, alcoholic hepatitis, autoimmune hepatitis, and even PBC. This meta-analysis deals with all *H*. *pylori* diagnostic techniques used in actual clinical practice. In addition, the studies included in this meta-analysis have similarities in the composition of sex and gender of the participants. There are low to moderate grades of heterogeneity and inconsistency across studies on PHG. Also, there was no detectable publication bias according to Begg’s rank correlation test and Egger’s regression test.

However, this study has some limitations and requires careful consideration when interpreting the results of the investigation. The total number of articles included in the meta-analysis was relatively small (13 articles). In addition, 5 out of 13 articles were case-control studies; therefore, there are only 8 cohort studies with high reliability. Because meta-analyses comprise already published articles, their conclusions are affected by the number and quality of the chosen studies. If more highly qualified original articles on the subject of the connection between *H*. *pylori* infection and PH-related GI complications are reported, more influential and reliable meta-analyses will be available. A meta-analysis that deals with the relationship between *H*. *pylori* infection and the risk of bleeding due to PH-related GI complications is also required because there are conflicting reports about the relationship between variceal bleeding and *H*. *pylori* infection [45,46].

In summary, our study demonstrated that gastric colonization of H. *pylori* patients with cirrhosis is unlikely to be attributed to the development of PH-associated GI complications. Knowing that *H*. *pylori* infection is not associated with PH-related GI complications in cirrhosis patients will help clinicians treat PH-related GI complications and H. pylori-induced gastritis in cirrhosis patients. Clinicians can treat PH-related GI complications in patients with cirrhosis, regardless of *H*. *pylori* infection.

## Supporting information

S1 Table Quality assessment of included case-control studies by the Newcastle-Ottawa scale. (DOCX)

S2 Table Quality assessment of included cohort studies by the Newcastle-Ottawa scale. (DOCX)

S3 Table Sensitivity analysis. (DOCX)

S4 Table PRISMA checklist. (DOCX)

S1 Text Supplementary methods. Search strategies for PubMed, EMBASE and the Cochrane Library.
(DOCX)
