# Supplementary material for: Helicobacter pylori infection is not associated with portal hypertension-related gastrointestinal complications: A meta-analysis
Source: PLoS One. 2022 Jan 21;17(1):e0261448. doi: 10.1371/journal.pone.0261448 (PMC8782498; doi:10.1371/journal.pone.0261448)
Supplement: S1 Table — (DOCX) [file pone.0261448.s001.docx]

**S1 Table. Quality assessment of included case-control studies by the Newcastle-Ottawa scale**

|  | **Selection** | | | | **Comparability** | **Exposure** | | |
| --- | --- | --- | --- | --- | --- | --- | --- | --- |
|  | Is the case definition adequate? | Representativeness of the cases | Selection of controls | Definition of controls | Comparability of cases and cohorts on the basis of the design or analysis | Ascertainment of exposure | Same method of ascertainment for cases and controls | Non-Response rate |
| Wu *et al*., 1995 | ★ | - | - | ★ | ★ | ★ | ★ | ★ |
| Bahnacy *et al*., 1997 | - | - | - | ★ | ★ | ★ | ★ | ★ |
| Chen *et al*., 2002 | ★ | - | - | ★ | ★ | ★ | ★ | - |
| Sathar *et al*., 2014 | ★ | - | - | ★ | ★ | ★ | ★ | ★ |
| Huang and Cui, 2017 | ★ | - | - | ★ | ★ | ★ | ★ | - |
