# Supplementary material for: Helicobacter pylori infection is not associated with portal hypertension-related gastrointestinal complications: A meta-analysis
Source: PLoS One. 2022 Jan 21;17(1):e0261448. doi: 10.1371/journal.pone.0261448 (PMC8782498; doi:10.1371/journal.pone.0261448)
Supplement: S2 Table — (DOCX) [file pone.0261448.s002.docx]

**S2 Table. Quality assessment of included cohort studies by the Newcastle-Ottawa scale**

|  | **Selection** | | | | **Comparability** | **Outcome** | | |
| --- | --- | --- | --- | --- | --- | --- | --- | --- |
|  | Representativeness of the exposed cohort | Selection of the non-exposed cohort | Ascertainment of exposure  ab | Demonstration that outcome of interest was not present at start of study | Comparability of cohorts on the basis of the design or analysis | Assessment of outcome  ab | Was follow-up long enough for outcomes to occur | Adequacy of follow-up of cohorts |
| Balan *et al*., 1996 | ★ | ★ | ★ | ★ | ★ | ★ | ★ | ★ |
| Tsai, 1998 | ★ | ★ | ★ | ★ | ★ | ★ | ★ | ★ |
| McCormick *et al*., 1999 | ★ | ★ | ★ | - | ★ | ★ | ★ | ★ |
| Yeh *et al*., 2001 | ★ | ★ | ★ | ★ | ★ | ★ | ★ | ★ |
| Arafa *et al.*, 2003 | ★ | ★ | ★ | ★ | ★ | ★ | ★ | ★ |
| Urso *et al*., 2006 | ★ | ★ | ★ | - | ★ | ★ | ★ | ★ |
| Abbas *et al*., 2014 | ★ | ★ | ★ | - | ★ | ★ | ★ | ★ |
| Abdel-Razik *et al*., 2020 | ★ | ★ | ★ | ★ | ★ | ★ | ★ | ★ |
