# Supplementary material for: Helicobacter pylori infection is not associated with portal hypertension-related gastrointestinal complications: A meta-analysis
Source: PLoS One. 2022 Jan 21;17(1):e0261448. doi: 10.1371/journal.pone.0261448 (PMC8782498; doi:10.1371/journal.pone.0261448)
Supplement: S3 Table — (DOCX) [file pone.0261448.s003.docx]

**S3 Table. Sensitivity analysis**

| Excluding literature | RR [95% CI] | |
| --- | --- | --- |
|  | Esophageal varix | Portal hypertensive gastropathy |
| None | 0.9613 [0.8687; 1.0639] | 1.0204 [0.9446; 1.1023] |
| Wu *et al*., 1995 | 0.9980 [0.9363; 1.0638] |  |
| Balan *et al*., 1996 |  | 1.0037 [0.9434; 1.0679] |
| Bahnacy *et al*., 1997 |  | 0.9978 [0.9379; 1.0615] |
| Tsai, 1998 | 0.9982 [0.9363; 1.0642] |  |
| McCormick *et al*., 1999 |  | 0.9983 [0.9388; 1.0616] |
| Yeh *et al*., 2001 | 0.9946 [0.9343; 1.0588] | 1.0003 [0.9403; 1.0642] |
| Chen *et al*., 2002 | 1.0003 [0.9394; 1.0650] |  |
| Arafa *et al.*, 2003 |  | 1.0012 [0.9409; 1.0654] |
| Urso *et al*., 2006 |  | 0.9887 [0.9226; 1.0594] |
| Abbas *et al*., 2014 | 1.0140 [0.9513; 1.0809] | 1.0111 [0.9410; 1.0865] |
| Sathar *et al*., 2014 |  | 0.9847 [0.9250; 1.0483] |
| Huang and Cui, 2017 |  | 0.9923 [0.9329; 1.0555] |
| Abdel-Razik *et al*., 2020 | 1.0000 [0.9385; 1.0655] | 0.9923 [0.9322; 1.0562] |
