# Supplementary material for: Helicobacter pylori infection is not associated with portal hypertension-related gastrointestinal complications: A meta-analysis
Source: PLoS One. 2022 Jan 21;17(1):e0261448. doi: 10.1371/journal.pone.0261448 (PMC8782498; doi:10.1371/journal.pone.0261448)
Supplement: S1 Text — Search strategies for PubMed, EMBASE and the Cochrane Library. (DOCX) [file pone.0261448.s005.docx]

**Supplementary Methods.**

Search strategies for PubMed, EMBASE and Cochrane Library were descripted.

PubMed: 752 results

("Helicobacter pylori"[All Fields] OR “H.pylori”[All Fields] OR “HP”[All Fields] OR "Campylobacter pylori"[All Fields] OR “C.pylori”[All Fields] OR “Helicobacter”[All Fields] OR “Campylobacter”[All Fields] OR “pylori”[All Fields] OR “pyloridis” [All Fields]) AND ("liver cirrhosis"[All Fields] OR “LC”[All Fields] OR "liver fibrosis"[All Fields] OR “hepatic fibrosis”[All Fields] OR "liver failure"[All Fields] OR “hepatic failure”[All Fields] OR “cirrho*” [All Fields] OR “varix” [All Fields] OR “variceal” [All Fields] OR “varicosis” [All Fields] OR "portal hypertensive gastropathy"[All Fields] OR “PHG” [All Fields] OR “congestive gastropathy”[All Fields]) AND ((“0001/01/01”[PDAT] : “2018/12/31”[PDAT]) AND “humans”[MeSH Terms])

EMBASE: 3,381 results

#1: ‘Helicobacter pylori’ OR ‘H.pylori’ OR ‘HP’ OR ‘Campylobacter pylori’ OR ‘C.pylori’ OR ‘Helicobacter’ OR ‘Campylobacter’ OR ‘pylori’ OR ‘pyloridis’ AND (‘liver cirrhosis’ OR ‘LC’ OR ‘liver fibrosis’ OR ‘hepatic fibrosis’ OR ‘liver failure’ OR ‘hepatic failure’ OR ‘cirrho*’ OR ‘varix’ OR ‘variceal’ OR ‘varicosis’ OR ‘portal hypertensive gastropathy’ OR ‘PHG’ OR ‘congestive gastropathy’): 4,062 results

#2: ‘human’/de: 21,762,856 results

#1 AND #2: 3,381 results

Cochrane Library: 296 results

#1 : (‘Helicobacter pylori’ OR ‘H.pylori’ OR ‘HP’ OR ‘Campylobacter pylori’ OR ‘C.pylori’ OR ‘Helicobacter’ OR ‘Campylobacter’ OR ‘pylori’ OR ‘pyloridis’) AND (‘liver cirrhosis’ OR ‘LC’ OR ‘liver fibrosis’ OR ‘hepatic fibrosis’ OR ‘liver failure’ OR ‘hepatic failure’ OR ‘cirrho*’ OR ‘varix’ OR ‘variceal’ OR ‘varicosis’ OR ‘portal hypertensive gastropathy’ OR ‘PHG’ OR ‘congestive gastropathy’): 355 results

#2. #1 Limits in Trials: 296 results
